# Reduction of Heterogeneous nuclear ribonucleoprotein A1 levels in retinal pigment epithelial cells induces inflammation and inhibits autophagy flux: pathology of age-related macular degeneration

**DOI:** 10.1016/j.bbrep.2025.102195

**Published:** 2025-08-07

**Authors:** Tomofumi Yatsu, Ayaka Nagata, Takuya Chiba, Yoshiki Miyata

**Affiliations:** a Faculty of Pharma-Sciences, Teikyo University, 2-11-1 Kaga, Itabashi-ku, Tokyo, 173-8605, Japan; b The Biomedical Gerontology Laboratory, Faculty of Human Sciences, Waseda University, 2-579-15, Mikajima, Tokorozawa-shi, 359-1192, Japan

**Keywords:** Autophagy, Age-related macular degeneration, Inflammation, Heterogeneous nuclear ribonucleoprotein A1

## Abstract

Heterogeneous nuclear ribonucleoprotein A1 (HNRNPA1) regulates RNA metabolism and inhibits various aging processes. It has also been reported as an inhibitor of inflammation; however, its role in the retina, particularly in retinal pigment epithelial (RPE) cells—a major source of inflammatory cytokines in the retina—remains unclear. Retinal inflammation is a key factor in the development of dry age-related macular degeneration (AMD), an age-related disease that can lead to blindness and currently lacks an established treatment. Therapeutic strategies are focused on preventing the suppression of autophagy, a precursor to inflammation. However, the factors regulating autophagy in RPE cells are not yet fully understood. In this study, we investigated the role of HNRNPA1 in RPE cells to evaluate its potential as a therapeutic target for dry AMD. *HNRNPA1* knockdown experiments were conducted, followed by RNA sequencing (RNA-seq) and Gene Ontology term analyses to elucidate the impact of HNRNPA1 reduction. The results revealed that reduced HNRNPA1 levels induced the increased expression of CXCL8 and IL1B, decreased autolysosome formation, and increased autophagosome formation, showing that HNRNPA1 reduction induces inflammation and suppressed autophagy, demonstrating its essential role in maintaining autophagy and mitigating inflammation under normal conditions. Furthermore, in an NaIO3-induced dryAMD model, RPE degeneration was accompanied by a reduction in HNRNPA1. These findings raise the possibility that decreased HNRNPA1 levels play a role in the onset and progression of dry AMD, and support the rationale for further exploring HNRNPA1 as a potential therapeutic target for this currently untreatable condition.

## Highlights

• *HNRNPA1* knockdown triggers inflammation and inhibits autophagy in ARPE-19 cells.
• HNRNPA1 reduction causes autophagosome accumulation, reduces autolysosome formation.
• Autophagy flux inhibitor chloroquine induces inflammation in ARPE-19 cells.
• HNRNPA1 is a potential therapeutic target for AMD.

## 1 Introduction

Heterogeneous nuclear ribonucleoprotein A1 (HNRNPA1) plays a critical role in various cellular processes, including regulation of gene expression through RNA metabolism, transcription, and multiple signaling pathways [1]. HNRNPA1 is an anti-senescence and autophagy-related factor, belonging to a family of proteins that bind to nascent pre-mRNA transcripts in the nucleus, thereby influencing their processing, transport, and translation [2,3]. Furthermore, HNRNPA1 is essential for cellular stress responses [1,4], and also contributes to the suppression of inflammation in different tissues and cells, highlighting its pivotal role in maintaining cellular homeostasis [4]. In particular, loss of HNRNPA1 has been linked to increased inflammation owing to increased levels of IL1B [5,6]. It is also involved in the expression of autophagy-related gene 6 (ATG6), known as Beclin-1 (BECN1), an essential factor for autophagy [3]. Autophagy inhibition, especially autophagosome accumulation, causes inflammation [7].

Persistent inflammation in the retina is associated with dry age-related macular degeneration (AMD), a leading cause of blindness. Dry AMD is characterized by the gradual degeneration of retinal pigment epithelial (RPE) cells and photoreceptors, resulting in geographic atrophy and central vision loss. If untreated, dry AMD can progress to wet AMD, causing more rapid vision loss. Therefore, controlling dry AMD is crucial for preventing blindness and improving patient outcomes. Inhibition of autophagy caused by oxidative stress and other factors, along with increased expression of *IL1B* and *CXCL8*, has been shown to play a critical role in its development[[8], [9], [10]]. However, the relationship between inflammation and HNRNPA1 in the retina and the function of HNRNPA1 in RPE cells remain unclear. We used RNA-seq to examine the function of HNRNPA1 in RPE, which are among the major producers of inflammatory cytokines in the retina. Generally, accumulation of autophagosomes leads to inflammation. To explore this relationship further, we examined the role of HNRNPA1 in autophagy regulation and its impact on inflammation in RPE cells.

## 2 Methods

### 2.1 Cell culture and transfection

ARPE-19 cells were maintained in Dulbecco's Modified Eagle Medium/Nutrient Mixture F-12 supplemented with 10 % fetal bovine serum (FBS, Thermo Fisher Scientific, Waltham, MA) and antibiotics (Thermo Fisher Scientific) at 37 °C in a humidified 5 % CO_2_ atmosphere. ARPE-19 cells were seeded in a six-well plate and incubated for 24 h. Subsequently, the cells were transfected with *HNRNPA1*-targeting or negative control (NC) small interfering RNAs (siRNAs) using Lipofectamine RNAimax (Thermo Fisher Scientific) following the protocol provided by the manufacturer. A dry AMD model was established via RPE degeneration by treating cells with 5, 10, 15, or 20 mM NaIO_3._ (Wako Pure Chemical Industries, Osaka, Japan) in 10 % FBS for 24 h. Experiments were performed using confluent ARPE-19 monolayers confirmed by phase-contrast microscopy.

### 2.2 RNA-seq

Total RNA was extracted using the NucleoSpin RNA kit (Takara Bio, Osaka, Japan) and quantified using NanoDrop, Qubit, and TapeStation. Samples (300 ng, RIN ≥7) underwent poly-A mRNA enrichment using the NEBNext Poly(A) mRNA Isolation Module, followed by cDNA synthesis and library preparation using the NEBNext Ultra II RNA Library Prep Kit. Libraries were barcode-labeled via 10-cycle PCR, pooled, and sequenced on a NovaSeq 6000 (Illumina) in 150-bp paired-end mode (∼20 M reads/sample). Reads were trimmed using Cutadapt, aligned to GRCh38 using HISAT2, and quantified using featureCounts [11]. Differentially expressed genes (DEGs) (|log2FC| > 1, p < 0.05) were identified using edgeR, and gene ontology (GO) analysis was conducted using goatools [12]. RNA-seq was performed at AZENTA, Japan. Additional molecular function (MF) and pathway analyses were performed using SRPlot [13].

### 2.3 RT-qPCR

Total RNA was isolated using ISOGEN II (Nippon Gene, Tokyo, Japan). Next, cDNA was synthesized using PrimeScript™ RT Master Mix (Perfect Real Time) (Takara Bio) following the protocol provided by the manufacturer. RT-qPCR was conducted on an Applied Biosystems 7500 Real-Time PCR System (Life Technologies; Thermo Fisher Scientific) using TB Green® Premix Ex Taq™ (Tli RNaseH Plus) (Takara Bio) as indicated by the manufacturer. The primer pairs used in this study are listed in Table S1.

### 2.4 Western blotting

Total protein was extracted using cell lysis buffer supplemented with a protease inhibitor cocktail (Cell Signaling Technology, NY, USA), and the protein concentration was measured using the BCA protein assay reagent (Thermo Fisher Scientific). Protein samples were separated through sodium dodecyl sulfate-polyacrylamide gel electrophoresis followed by immunoblotting using rabbit anti- Light chain 3 (LC3) (Cell Signaling Technology, Danvers, MA, USA), anti-HNRNPA1 (Cell Signaling Technology) and anti-ATG5 (Cell Signaling Technology) as primary antibodies, and mouse anti-β-actin, which was used as the loading control. Horseradish peroxidase–conjugated anti-rabbit IgG and anti-mouse IgG (Cell Signaling Technology) were used as secondary antibodies. Protein bands were visualized, and signals were captured using the ECL™ Prime Western Blotting Detection Reagent and AI680 (Global Life Sciences Technologies Japan, Tokyo, Japan), respectively. Band density was analyzed using ImageJ software (v1.54d, National Institutes of Health).

### 2.5 Autophagosome and autolysosome detection

ARPE-19 cells were seeded onto microslides and treated with 50 μM chloroquine (CQ; Tokyo Chemical Industry, Tokyo, Japan), an autophagy inhibitor, for 24 h. Alternatively, the cells were transfected with siNC or siHNRNPA1 to induce knockdown and incubated for 3 days, or subjected to a 6-h treatment with 20 mM NaIO3. ARPE-19 cells were stained with DALGreen (DOJINDO LABORATORIES) or DAPRed (DOJINDO LABORATORIES) to detect autolysosomes and autophagosomes, respectively, following the protocol provided by the manufacturer. The cells were observed using a fluorescent microscope (BZ-X810, KEYENCE, Osaka, Japan).

### 2.6 Statistical analysis

The results are expressed as mean ± S.D. The probability of statistical differences between the experimental groups was determined using Welch's *t*-test and Dunnett’s test. All statistical analyses were performed using GraphPad Prism9 (GraphPad Software, San Diego, CA). Statistical significance was set at p < 0.05.

## 3 Results

### *3.1* *RNA-seq analysis in HNRNPA1 knockdown ARPE-19* cells

**Fig. 1:**
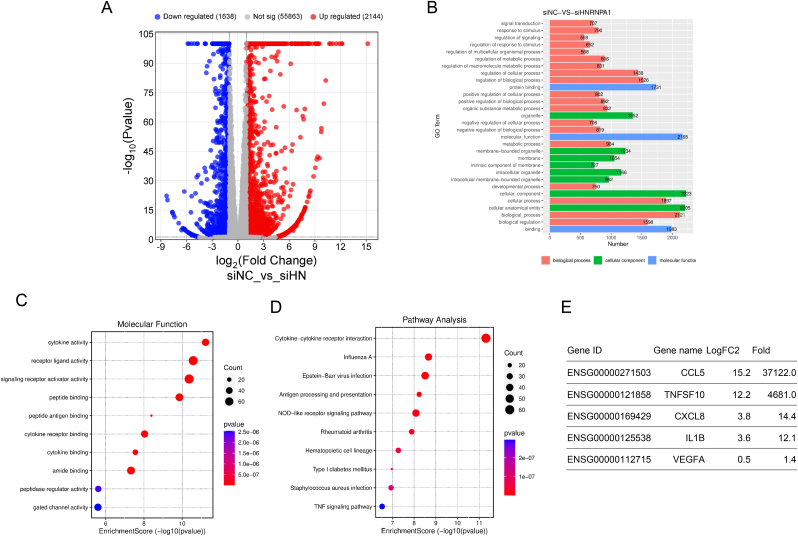
Inflammation induction by *HNRNPA1* knockdown in ARPE-19 cells. ARPE-19 cells were transfected with *HNRNPA1*-targeting siRNA or siNC; 3 d after transfection, mRNA was extracted. The transcriptome was analyzed using NGS. (A) Volcano plot showing gene expression levels and P values. (B) Gene ontology (GO) term analysis bar plot. (C) Dot plot of the molecular function of 1331 upregulated genes grouped in GO:0005575. (D) Dot plot of the pathway analysis of 1331 upregulated genes grouped in GO:0005575. (E) Expression of AMD-related genes.

To elucidate the role of HNRNPA1 in RPE, one of the primary inflammatory cytokine-expressing cells, *HNRNPA1* was knocked down in ARPE-19 cells, followed by RNA-seq analysis. The volcano plot is presented in Fig. 1A. A GO term analysis of these genes revealed the most frequent alterations in 2223 genes grouped in the GO:0005575 cellular component, followed by 2205 genes in GO:0110165 cellular anatomical entity (Fig. 1B). Among the 2223 genes in GO:0005575, 1331 were upregulated. To investigate the changes in signaling associated with these altered gene expression patterns, pathway and molecular function analyses of these 1331 genes were conducted; cytokine activity and cytokine–cytokine receptor interactions exhibited the highest enrichment scores (Fig. 1C and D). The relative expression levels of *IL1B* and *CXCL8*, two major proinflammatory cytokines involved in RPE inflammation, were increased by 14.4-fold and 12.1-fold, respectively, as shown in Fig. 1E. C–C motif chemokine 5 (CCL5), which has been reported as an AMD-related chemokine [14], was upregulated by 37,122-fold as a result of the reduction of HNRNPA1 (Fig. 1E). Moreover, *HNRNPA1* knockdown increased the expression of tumor necrosis factor superfamily member 10 (TNFSF10) (Fig. 1E), which has been reported as one of the therapeutic targets for retinal inflammation [15]. On the other hand, VEGFA, which encodes vascular endothelial growth factor, a therapeutic target in wet AMD, an advanced form of AMD, was not strongly upregulated (Fig. 1E). These results indicate that *HNRNPA1* knockdown in ARPE-19 cells induces the expression of genes associated with inflammation.

### 3.2 HNRNPA1 knockdown caused autophagosome accumulation in ARPE-19 cells

**Fig. 2:**
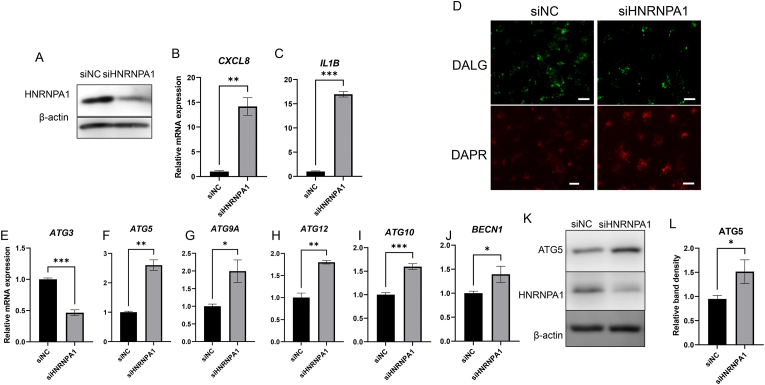
*HNRNPA1* knockdown caused autophagosome accumulation in ARPE-19 cells. ARPE-19 cells were transfected with *HNRNPA1*-targeting siRNA or siNC; 3 d after transfection, (A, K) proteins were detected through immunoblotting using antibodies against (A, K) HNRNPA1, (K) ATG5, with β-actin used as the loading control. (B, C) The mRNA expression levels of (B) *CXCL8* and (C) *IL1B* were measured using RT-qPCR. (D) The cells were stained with DAPRed as an autophagosome (red) detection reagent and DALGreen as an autolysosome (green) detection reagent. (E–J) RT-qPCR revealed the gene expression levels of (E) *ATG3*, (F) *ATG5*, (G) *ATG9A*, (H) *ATG10*, (I) *ATG12*, and (J) *BECN1*. (L) ATG5 band intensity was quantified and normalized with β-actin (n = 3–4, mean ± S.D.). ∗p < 0.05, ∗∗p < 0.01, ∗∗∗p < 0.001 vs. siNC. Scale bar indicates 100 μm.

Using Western blot analysis and qPCR, we revealed that *HNRNPA1* knockdown (Fig. 2A) induced 14.2- and 17.0-fold increases in *CXCL8* (Fig. 2B) and *IL1B* (Fig. 2C) gene expression, respectively, compared with siNC-transfected cells, indicating that *HNRNPA1* knockdown triggers inflammation in ARPE-19 cells. Notably, both IL1B and CXCL8, the upregulated cytokines, are known to play significant roles in the development and progression of dry AMD [9]. Generally, one of the main mechanisms of inflammatory cytokine upregulation is autophagosome accumulation [16]. Therefore, we examined the effects of *HNRNPA1* knockdown on autophagosome accumulation. DALGreen detects autolysosomes and emits green fluorescence, whereas DAPRed detects autophagosomes and emits red fluorescence [17]. In *HNRNPA1*-knockdown cells, the green fluorescence emitted by DALGreen was lower than that in siNC-transfected cells, whereas red fluorescence emitted by DAPRed was increased, indicating autophagosome accumulation. (Fig. 2D). Autophagy acceleration involves the increase of both autophagosomes and autolysosomes. However, during autophagosome accumulation, only autophagosomes increase, whereas autolysosomes do not [18]. Overall, these results indicate that HNRNPA1 may modulate homeostasis of autophagosome accumulation.

To elucidate the mechanism by which the reduction in HNRNPA1 levels induces autophagosome accumulation, we investigated the effects of lower levels of HNRNPA1 on the expression levels of genes encoding autophagosome formation–accelerating factors. Specifically, ARPE-19 cells were transfected with siHNRNPA1 or siNC, and RNA expression levels of the autophagosome formation–accelerating factors *ATG3*, *ATG5*, *ATG9A*, *ATG10*, *ATG12*, and *BECN1* were measured using RT-qPCR [19]. The results showed that *ATG3* gene expression levels in siHNRNPA1-transfected ARPE-19 cells were lower than those in siNC-transfected cells (Fig. 2E). Autophagosomes are divided into mature autophagosomes, which surround insoluble proteins, and immature autophagosomes, which do not surround insoluble proteins. ATG3 is a factor involved in autophagosome maturation [20]. Therefore, it was suggested that the reduction in ATG3 by *HNRNPA1* knockdown may suppress autophagosome maturation. Contrastingly, *ATG5*, *ATG7*, *ATG9A*, *ATG10*, *ATG12*, and *BECN1* gene expression levels were higher in siHNRNPA1-transfected cells than those in siNC-transfected ones (Fig. 2F–J). These are factors that promote the supply of membrane components to immature autophagosomes [19]. ATG5, a key component of autophagy, regulates the formation of the autophagosome, a hallmark of autophagy [20]. Subsequently, we investigated the protein expression of ATG5 and found that it was significantly upregulated upon HNRNPA1 knockdown (Fig. 2K and L). Taken together, these findings indicate that *HNRNPA1* knockdown induces autophagosome accumulation by downregulating the autophagosome maturation factor ATG3 and promotes the formation of immature autophagosomes by upregulating ATG5, ATG9A, ATG10, and ATG12. These results suggest that HNRNPA1 contributes to the inhibition of autophagosome accumulation.

### 3.3 Inhibition of CQ-inducible LC3 upregulation by HNRNPA1 knockdown in ARPE-19 cells

The results described above have clearly established that a reduction in HNRNPA1 within ARPE-19 cells induces autophagosome accumulation and upregulates the gene expression of *IL1B* and *CXCL8*. To validate whether autophagy suppression with autophagosome accumulation directly induces the expression of inflammatory cytokines in RPE, we investigated the effects of CQ, which is an autophagosome accumulation inducer, on ARPE-19 cells using RT-qPCR.

**Fig. 3:**
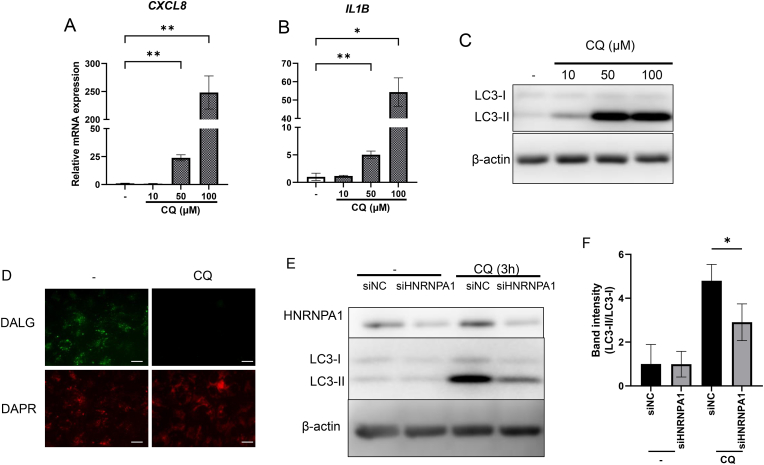
Inhibition of chloroquine (CQ)-inducible LC3 upregulation by *HNRNPA1* knockdown in ARPE-19 cells ARPE-19 cells were treated with 10, 50, or 100 μM CQ or 0.1% DMSO as a solvent control; the cells were (A–C) harvested 24 h after treatment. The mRNA expression levels of (A) *CXCL8* and (B) *IL1B* were measured using RT-qPCR. (C) Proteins were detected through immunoblotting using antibodies against LC3, with β-actin used as the loading control. (D) The cells were stained with DAP Red as an autophagosome (red) detection reagent and DAL Green as an autolysosome (green) detection reagent. (E, F) The cells were treated with solvent control or 50 μM CQ 3 d after transfection; 3 h after CQ treatment, the cells were harvested. The protein expression levels of HNRNPA1, LC3-I, LC3-II, and β-actin (loading control) were evaluated using western blotting. (F) Band intensity was quantified. The results were evaluated with ratio of LC3-II/LC3-I which are expressed as mean ± S.D. (n = 3–4) ∗p < 0.05, ∗∗p < 0.01 vs. solvent control. Scale bar indicates 100 μm.

The results demonstrated that, as observed with *HNRNPA1* knockdown, gene expression of *CXCL8* and *IL1B* increased with CQ treatment in a concentration-dependent manner as expected (Fig. 3A and B). Additionally, conversion of LC3-I to LC3-II which is evaluated by ration of LC3-II/LC3-I, a key autophagy marker that reflects mature autophagosome formation and turnover [19], increased in a CQ concentration-dependent manner, indicating suppressed turnover by autophagy as expected (Fig. 3C). This result suggests that 50 μM CQ induce autophagosome accumulation. Furthermore, to evaluate CQ-induced autophagosome accumulation, DALGreen and DAPRed were used to detect autolysosomes and autophagosomes, respectively, in ARPE-19 cells. The results indicated that autolysosomes were strongly decreased and autophagosomes including immature autophagosomes were increased by CQ (50 μM) treatment in ARPE-19 cells as expected (Fig. 3D). These findings suggest that accumulation of autophagosomes induces inflammation-related genes *IL1B* and *CXCL8* in ARPE-19 cells.

Generally, the inhibition of autophagic flux increases the accumulation of insoluble proteins and induces inflammation [16,21]. Autophagy significantly contributes to cellular homeostasis, and its dysfunction leads to chronic inflammation and cellular dysfunction, such as that associated with AMD [22].

Furthermore, the effect of HNRNPA1 on autophagy was examined using Western blot analysis. Because CQ induces autophagosome accumulation, it was used to evaluate the stage of autophagosome formation. The results revealed that ARPE-19 cells transfected with siHNRNPA1 and treated with CQ had significantly lower ratio of LC3-II/LC3-I expression than ARPE-19 cells transfected with siNC and treated with CQ, indicating that autophagosome maturation was suppressed by *HNRNPA1* knockdown (Fig. 3B and C). Under conditions where autophagy is suppressed at the initiation stage, the LC3-II/LC3-I ratio fails to increase or may even decrease upon CQ treatment, indicating reduced autophagosome formation and overall autophagic flux [23]. Decreased LC3 is also a phenomenon observed in the RPE of patients with dry AMD [24]. CQ treatment also increased HNRNPA1 expression, which may be feedback to autophagy repression (data not shown). These suggest that *HNRNPA1* knockdown causes induction of inflammation-related genes through the accumulation of immature autophagosomes. In the absence of autolysosome inhibitors, LC3-II is rapidly degraded; therefore, it is not recommended to evaluate changes in the LC3 ratio under such conditions (23). Specifically, the inhibition of autophagy flux by HNRNPA1 knockdown is mediated by suppression of LC3 maturation, due to increased ATG5 and decreased ATG3 expression, resulting in reduced LC3-II formation. Under CQ treatment, degradation of LC3-II is blocked, which increases the LC3-II/I ratio in the siNC group. However, in the siHNRNPA1 group, LC3-II formation is suppressed, resulting in a lower LC3-II/I ratio despite the presence of CQ.

### 3.4 Dry AMD model of RPE degeneration decreased HNRNPA1 in ARPE-19 cells

**Fig. 4:**
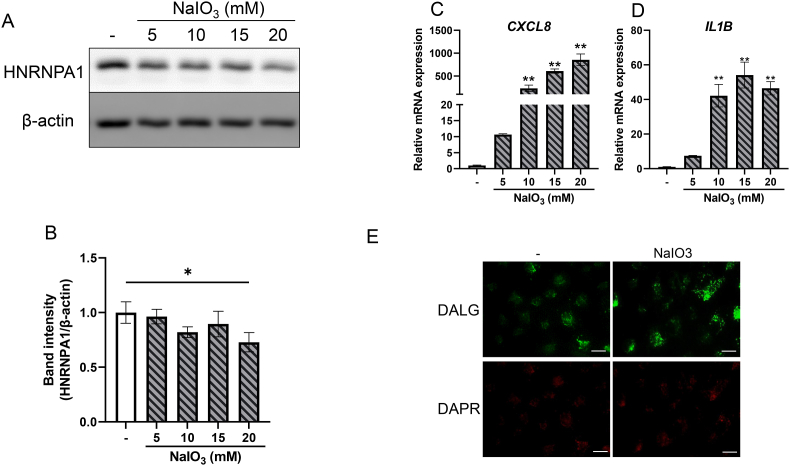
Reduction of HNRNPA1 in NaIO3-inducible RPE degeneration as a dry AMD model. (A–D) ARPE-19 cells were treated with 5, 10, 15, or 20 mM NaIO3. (A, B) The cells were harvested 24 h after treatment. Proteins were detected through immunoblotting using antibodies against HNRNPA1 with β-actin used as the loading control. (B) The band density was quantified. (C, D) The mRNA expression levels of (C) *CXCL8* and (D) *IL1B* were measured using RT qPCR. The results were normalized to β-actin. (E) The cells were stained with DAP Red, an autophagosome (red) detection reagent, and DAL Green, an autolysosome (green) detection reagent, 6 h after treatment with 20 mM NaIO3. Results are expressed as mean ± S.D. (n = 3–4) ∗p < 0.05, ∗∗p < 0.01 vs control. Scale bar indicates 100 μm.

As autophagosome accumulation was observed in patients with dry AMD [25], *HNRNPA1* knockdown may reproduce this phenomenon. Hence, we propose HNRNPA1 to be a novel target involved in autophagy regulation and constitutive anti-inflammation for the treatment of dry AMD. We therefore finally sought to clarify how HNRNPA1 is involved in dry AMD. We used ARPE-19 cells in the NaIO3-inducible RPE degeneration model as a dry AMD model and analyzed RPE degeneration effects on the expression of HNRNPA1 [25]. We observed that the expression level of the HNRNPA1 was decreased in ARPE-19 cells treated with NaIO3 for 24 h in a dose–dependent manner (Fig. 4A and B). Further qPCR analysis of the effect of NaIO3 treatment on the inflammatory cytokine–encoding genes showed that the expression of *CXCL8* (Fig. 4C) and *IL1B* (Fig. 4D) was increased in a dose-dependent manner, indicating inflammation similar to that induced by *HNRNPA1* knockdown. Finally, we evaluated the effect on autophagy using DALGreen and DAPRed as probes for autolysosomes and autophagosomes, respectively. We found that, NaIO3 treatment increased autolysosome, but not autophagosome content (Fig. 4E), suggesting that autolysosome formation is induced in RPE degeneration. Acute increases in oxidative stress are known to promote autophagosome and autolysosome formation, while chronic increases in oxidative stress lead to autophagosome accumulation [22]. As NaIO3 also induces oxidative stress [25], the results in Fig. 4E may be due to an acute increase in oxidative stress. The results seen with *HNRNPA1* knockdown in this study may be similar to those in a chronic oxidative stress exposure model [24].

The results of this study revealed that a reduction in HNRNPA1 expression levels induces inflammation and autophagosome accumulation. Furthermore, it was elucidated that during NaIO3-induced RPE degeneration, the downregulation of HNRNPA1 may contribute to inflammation.

## 4 Discussions

This study elucidates the critical role of HNRNPA1 in ARPE-19 cells and demonstrates that its downregulation promotes inflammation as evidenced by RNA-seq analysis. Promotion of inflammation and accumulation of autophagosomes in the RPE have been reported to be observed in the early stages of dry AMD onset [22]. It is similar to the phenomenon by HNRNPA1 downregulation in ARPE-19 cells in our study. Chronic reactive oxygen species exposure models with CQ treatment have been reported to exhibit autophagosome accumulation owing to reduced LC3 levels [24], similar to what was observed with HNRNPA1 knockdown with CQ treatment in this study. These results indicate that suppression of HNRNPA1 expression may be a one of the critical mechanism underlying RPE inflammation.

To our knowledge, this is the first study to establish a relationship between HNRNPA1, inflammation, and autophagy in ARPE-19 cells. RNA-seq analysis revealed that a low level of HNRNPA1 leads to the induction of inflammation-related genes. Meanwhile, when we examined biological processes using RNA-seq analysis, DEGs in regulation of autophagy (GO:0010506) revealed increased expression of genes such as LAMP3, TRIM14, TRIM22 and HK2 (Table S2).In particular, LAMP3 has been reported as an inhibitor of autolysosome formation [26]. Therefore, factors beyond those examined in Fig. 2E–J also are likely involved in the accumulation of autophagosomes induced by *HNRNPA1* knockdown.

Inflammation was observed both in the dry AMD models employed in this study along with decreased HNRNPA1 levels, as well as in the *HNRNPA1* knockdown experiment. This highlights HNRNPA1 downregulation as a potentially critical mechanism in the development of dry AMD. This study demonstrated that *HNRNPA1* knockdown significantly increases the expression of major pro-inflammatory cytokines in RPE, such as IL1B and CXCL8. These findings support previous reports suggesting the involvement of HNRNPA1 in inflammation suppression [5,6]. However, the specific molecular mechanisms through which HNRNPA1 inhibits certain inflammatory signaling pathways, such as NF-κB or STAT3 activation, require further investigation.

The NaIO3-induced RPE degeneration model revealed a distinct autophagy profile compared with that in *HNRNPA1* knockdown. While *HNRNPA1* knockdown resulted in autophagosome accumulation (Fig. 2D), NaIO3 treatment increased autolysosome formation without evident autophagosome accumulation (Fig. 4E). This suggests that NaIO3 induces a short-term, acute autophagic response, likely driven by oxidative stress, whereas *HNRNPA1* knockdown disrupts autophagy in a more chronic manner, mimicking the long-term autophagy dysfunction observed in patients with dry AMD. Additionally, NaIO3 treatment reduced HNRNPA1 protein levels (Fig. 4A and B) and concurrently upregulated *IL1B* and *CXCL8* expression (Fig. 4C and D), further supporting the connection between HNRNPA1 downregulation and inflammation.

The observed inflammation induction and autophagosome accumulation in our models support the hypothesis that HNRNPA1 downregulation is a key driver of dry AMD pathogenesis.

However, this study is limited to in *vitro* knockdown experiments. Further investigations involving HNRNPA1 overexpression in dry AMD *in vitro* models, RPE cells, and animal models are essential to fully elucidate the role of HNRNPA1 in the phenomenon of dry AMD. It is also necessary to further investigate the expression of HNRNPA1 in dry AMD model animals and patient with dry AMD. Additionally, considering the multifactorial nature of dry AMD, it is unlikely that HNRNPA1 alone accounts for the entire pathogenesis of the disease. Future studies should focus on clarifying whether HNRNPA1 is a potential therapeutic target in light of these considerations. Moreover, the primary targets of HNRNPA1 have not yet been identified in this study. Therefore, further investigation at earlier time points after knockdown will be necessary to elucidate whether the observed effects on autophagy are direct or secondary. And more, future studies should include DALG/DAPR staining in CQ-treated siHNRNPA1 cells to further clarify the mechanisms by which HNRNPA1 knockdown modulates autophagic flux under lysosomal inhibition.

Given that HNRNPA1 is a ubiquitously expressed RNA-binding protein involved in multiple essential cellular processes, systemic modulation of its expression could lead to off-target effects. Therefore, future therapeutic strategies should consider cell type-specific or localized delivery methods to minimize potential side effects. Our current findings provide a mechanistic basis for such approaches in the context of dry AMD.

In conclusion, our findings suggest that downregulation of HNRNPA1 may contribute to the pathogenesis of dry AMD, and provide preliminary insights that could inform the development of future preventive or therapeutic strategies targeting HNRNPA1.

## CRediT authorship contribution statement

**Tomofumi Yatsu:** Writing – original draft, Software, Resources, Methodology, Investigation, Funding acquisition, Formal analysis, Data curation. **Ayaka Nagata:** Formal analysis, Data curation. **Takuya Chiba:** Writing – review & editing, Conceptualization. **Yoshiki Miyata:** Project administration.

## Funding sources

This work was supported by JSPS KAKENHI (grant number JP22K14847 to T.Y.).

## Declaration of competing interest

The authors declare that they have no known competing financial interests or personal relationships that could have appeared to influence the work reported in this paper.

## Data Availability

Data will be made available on request.
