# Supplementary material for: Reduction of Heterogeneous nuclear ribonucleoprotein A1 levels in retinal pigment epithelial cells induces inflammation and inhibits autophagy flux: pathology of age-related macular degeneration
Source: Biochem Biophys Rep. 2025 Aug 7;43:102195. doi: 10.1016/j.bbrep.2025.102195 (PMC12354792; doi:10.1016/j.bbrep.2025.102195)
Supplement: Multimedia component 1 [file mmc1.docx]

**Supplemental Tables.**


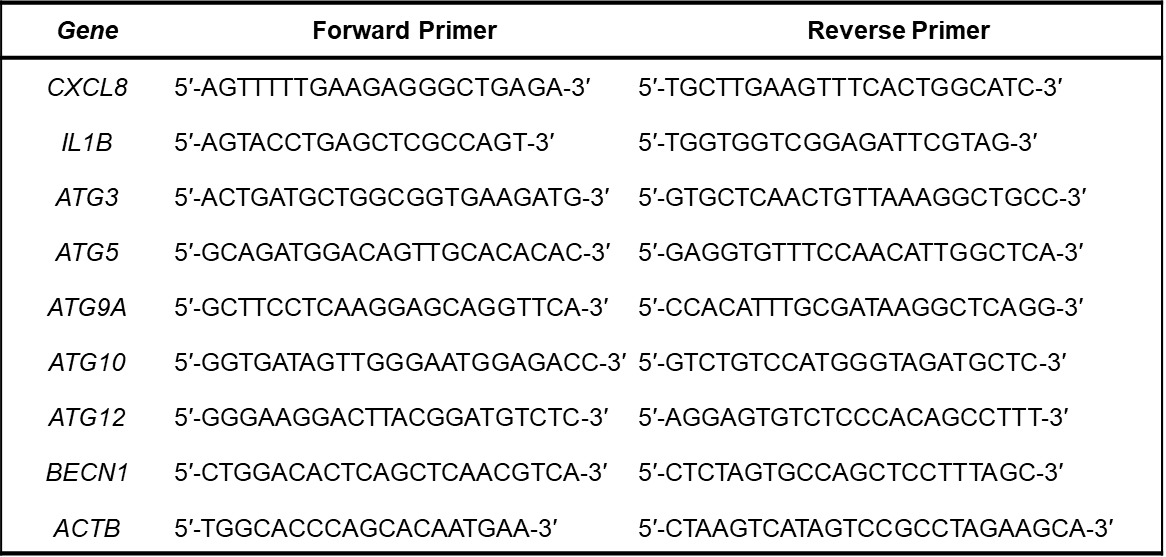
Supplemental Table 1. Primer list

| Gene ID | Gene name | LogFC2 | Fold |
| --- | --- | --- | --- |
| ENSG00000078081 | LAMP3 | 7.46 | 175.7 |
| ENSG00000106785 | TRIM14 | 2.35 | 5.1 |
| ENSG00000132274 | TRIM22 | 1.80 | 3.5 |
| ENSG00000159399 | HK2 | 1.77 | 3.4 |

Supplemental Table 2. DEG of biological process: regulation of autophagy (GO:0010506)
